# Molecular Access Engineering for Microbial Biocatalysis: from Enzyme Tunnels to Microbial Cell Factories

**DOI:** 10.4014/jmb.2607.07018

**Published:** 2026-07-23

**Authors:** Suk Min Kim

**Affiliations:** Department of Biotechnology, The Catholic University of Korea, Gyeonggi-do 14662, Republic of Korea

**Keywords:** Molecular access engineering, Molecular accessibility, Microbial biocatalysis, Enzyme engineering, Whole-cell biocatalysis, Microbial cell factories

## Abstract

Microbial biocatalysis spans biological platforms ranging from purified enzymes and multienzyme assemblies to electroenzymatic systems, whole-cell biocatalysts, and genetically programmable microbial cell factories. Yet despite their biological diversity, functional biocatalytic performance is often influenced by both catalytic activity and molecular accessibility. This recurring constraint highlights molecular accessibility as a common engineering consideration across microbial biocatalysis. Accordingly, this Review presents the concept of molecular access engineering as the rational control of molecular accessibility to improve functional biocatalytic performance across biological scales. The concept encompasses three complementary strategies governing molecular entry, intermediate transfer, and molecular exchange throughout biological systems. Examples from gas-converting enzymes, multienzyme assemblies, electroenzymatic systems, catalytic cascades, whole-cell biocatalysts, and microbial cell factories illustrate how engineering molecular accessibility can improve catalytic robustness, pathway efficiency, biological compatibility, and systems-level productivity. These examples therefore suggest that molecular access engineering has the potential to broaden the design space for microbial biocatalysis by complementing catalytic engineering.

## From Catalytic Activity to Molecular Accessibility

Microbial biocatalysis has evolved far beyond isolated enzymes to encompass increasingly integrated systems, including multienzyme assemblies, electroenzymatic platforms, whole-cell biocatalysts, and genetically programmable microbial cell factories [1-3]. These advances have transformed sustainable biomanufacturing by enabling the conversion of renewable feedstocks into a broad range of biobased products [2, 4, 5], including sustainable aviation fuels, biodegradable polymers, and high-value therapeutics. As these systems become more integrated and complex, the focus of biocatalyst design has increasingly shifted from optimizing individual enzymes [3, 6] to coordinating molecular processes that influence system-level performance [7].

Over the past three decades, biocatalyst development has been driven by active-site engineering, directed evolution, de novo enzyme design, metabolic engineering, and synthetic biology [1, 8-11]. These advances have enabled industrial-scale bioproduction, selective biotransformations [12, 13], and sophisticated biosynthetic pathways comprising dozens of catalytic reactions [14]. Yet catalytic activity alone rarely guarantees optimal biocatalytic performance, despite continuous improvements in catalytic engineering [15, 16]. Performance increasingly reflects how efficiently molecules are delivered [17], exchanged [18], and coordinated within integrated systems [19]. Meeting this challenge requires strategies that extend beyond catalytic optimization [20-22].

**Fig. 1:**
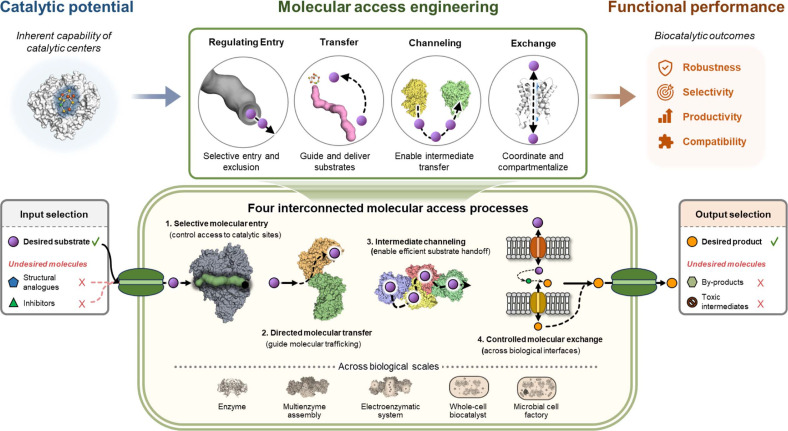
Molecular access engineering for microbial biocatalysis. Catalytic engineering defines the intrinsic chemical capability of catalytic centers, whereas molecular access engineering shapes how efficiently that capability is translated into functional biocatalytic performance. The framework comprises three engineering strategies (molecular entry, molecular transfer, and molecular exchange), illustrated through four representative molecular-access processes: (1) selective molecular entry, (2) directed molecular transfer, (3) intermediate channeling, and (4) controlled molecular exchange. Intermediate channeling is presented as a specialized molecular-transfer process within the molecular transfer strategy rather than as a separate engineering strategy. Across biological scales, this framework improves robustness, selectivity, productivity, and functional compatibility from enzymes and multienzyme assemblies to electroenzymatic systems, whole-cell biocatalysts, and microbial cell factories.

Despite their diverse biological organization, microbial biocatalytic systems often share a common physicochemical constraint that controls access to catalytic functions. Examples include gas diffusion through enzyme tunnels [23, 24], substrate channeling [18], membrane transport [17], and metabolic organization [3, 7], which have traditionally been investigated independently. Although these mechanisms appear distinct, these processes can be viewed as regulating a common underlying property, molecular accessibility. As illustrated in Fig. 1, this shared property may provide the conceptual link connecting seemingly disparate microbial biocatalytic systems.

In this Review, we propose molecular access engineering as a conceptual framework for microbial biocatalysis. Rather than replacing established concepts such as diffusion, transport engineering, or substrate channeling, molecular access engineering brings them together through the shared engineering objective of improving molecular accessibility. Tunnel engineering, transporter engineering, and mass-transfer engineering represent complementary approaches that address distinct aspects of molecular accessibility. This framework positions molecular accessibility as a complementary design dimension alongside catalytic engineering and encompasses three strategies that regulate molecular entry, transfer, and exchange. These strategies are realized through distinct molecular-access processes across different biological systems (Fig. 1). Among these processes, intermediate channeling represents a specialized mechanism of molecular transfer that facilitates the preferential transfer of intermediates between sequential catalytic sites.

## Molecular Accessibility across Microbial Biocatalytic Platforms

Remarkable advances in catalytic engineering have substantially improved microbial biocatalysis [1, 8, 20]. Yet improvements in catalytic chemistry do not always translate into proportional gains in overall biocatalytic performance [12, 22]. Beyond catalytic chemistry, biological catalysis depends on molecular accessibility, which helps determine whether molecules reach, interact with, and leave catalytic sites [23, 25]. Consequently, catalysts with comparable catalytic activities can exhibit markedly different performance [24, 26] because molecular accessibility shapes how effectively catalytic potential is translated into functional performance. More broadly, this property contributes to functional compatibility among catalytic processes across enzymes [23], multienzyme assemblies [18], biological interfaces [17], and whole-cell systems [3]. This distinction is exemplified by carbon monoxide dehydrogenases (CODHs) [27, 28], which exhibit turnover frequencies exceeding 30,000 s^–1^ [29] yet undergo rapid inactivation upon O_2_ exposure [30, 31] because O_2_ accessibility, rather than catalytic turnover, limits performance.

Across diverse biological architectures, microbial biocatalytic platforms repeatedly solve the same molecular challenges [12, 19], including substrate entry [23, 25], intermediate transfer [18], inhibitor exclusion [24], product release [23, 25], and molecular exchange [3, 17]. Although implemented through different biological mechanisms, they often reflect a common functional requirement of efficient and selective molecular accessibility. These recurring processes complement catalytic mechanisms by influencing how efficiently catalytic potential is translated into functional performance. This shared property arises regardless of the underlying catalytic mechanism, suggesting that the concept of molecular accessibility may be transferable across otherwise unrelated microbial biocatalytic platforms. Molecular accessibility thus provides a conceptual basis for molecular access engineering.

This general concept is realized through distinct biological mechanisms across microbial biocatalytic platforms. At the protein level, molecular accessibility is regulated by substrate tunnels [23, 32], gating residues [25, 33], and transport pathways that connect protein surfaces to buried catalytic sites [26, 32, 33]. In multienzyme assemblies, coordination is achieved through substrate channeling [18], including tunnel-mediated intermediate transfer, and catalytic communication [7]. A representative example is the approximately 70 Å internal tunnel connecting CODH and acetyl-CoA synthase (ACS), enabling direct CO transfer between the two active sites [34-36]. Electroenzymatic systems likewise depend on efficient electron delivery to catalytic sites [37-39], whereas whole-cell biocatalysts and microbial cell factories regulate molecular accessibility through membrane transport [17], intracellular organization [3, 7], and metabolic coordination [3, 19], with transport often becoming rate-limiting despite highly active intracellular enzymes [17, 22]. Despite operating at different biological scales, these systems share a common engineering objective of optimizing molecular access to enhance biocatalytic performance. Across diverse biocatalytic systems, molecular accessibility can be viewed as a transferable engineering principle that links catalytic potential to biocatalytic performance, thereby representing a distinct design dimension for future microbial biocatalysis. Table 1 summarizes this conceptual framework by presenting representative molecular access-engineering strategies across microbial biocatalytic platforms.

## Engineering Molecular Accessibility for Improved Microbial Biocatalysis

### Improve biocatalytic robustness by regulating molecular entry

Catalytic robustness frequently limits the practical implementation of microbial biocatalysts under industrial conditions [15, 22, 40, 41]. Many enzymes possess highly efficient catalytic machinery yet remain vulnerable to competing molecules, environmental fluctuations, or irreversible inactivation during operation [15, 20, 28, 40-42]. In such cases, biocatalytic performance is often constrained less by catalytic turnover than by molecular accessibility to catalytic sites [23, 24, 26, 43-45]. Engineering molecular entry thus provides a complementary strategy for enhancing robustness while preserving intrinsic catalytic activity.

Gas-converting metalloenzymes provide compelling examples of this principle [28, 46-48]. CODH contains a deeply buried Ni–Fe–S C-cluster [27, 47, 49] accessed through branched gas-access tunnels that mediate substrate CO delivery [49] and inhibitory O_2_ diffusion [30, 31]. Oxygen sensitivity therefore reflects both intrinsic catalytic-center reactivity [28, 47] and insufficient discrimination between productive CO entry [49] and nonproductive O_2_ entry [30, 31]. Bottleneck substitutions (A559W, V610H, and A559W/V610H) selectively reduced O_2_ accessibility while maintaining CO diffusion [30, 31], whereas multilayer tunnel sealing further enhanced aerobic robustness without altering catalytic architecture. These findings show that regulating molecular entry improves robustness without redesigning the catalytic center.

This principle extends beyond gas-converting metalloenzymes [23, 25, 43, 44]. In hydrogenases, narrowing gas tunnels decreases O_2_ accessibility while preserving H2 transport [50, 51], and naturally oxygen-tolerant enzymes possess narrower gas tunnels than oxygen-sensitive homologs [51-53], suggesting evolutionary optimization of molecular entry. Likewise, substrate-tunnel engineering in cytochrome P450 monooxygenases increased activity toward bulky substrates (F79A) and reshaped substrate specificity (F173V) [54], while access-tunnel engineering in P450 BM3 improved coupling efficiency from 31% to >50% without altering catalytic residues [55]. Similarly, tunnel redesign in haloalkane dehalogenases through mutations such as L177W and F246A accelerated product release [56, 57], broadened substrate scope [26], and improved catalytic efficiency by facilitating substrate access and product release [23, 43]. These findings support molecular entry as a broadly applicable strategy to improve biocatalytic robustness by optimizing molecular accessibility while preserving the intrinsic catalytic machinery (Fig. 1).

### Improve pathway efficiency by coordinating molecular transfer

While regulating molecular entry enhances individual enzyme performance [23, 26, 43], multienzyme assemblies [7, 58], catalytic cascades [18, 59], and synthetic metabolic pathways [6, 19, 60] shift the engineering challenge toward coordinated molecular transfer. This engineering objective can be achieved through diverse biological processes, among which substrate channeling represents one of the best-characterized mechanisms of molecular transfer. By minimizing intermediate loss while coordinating sequential catalytic reactions, substrate channeling enhances pathway efficiency. Consequently, overall pathway performance is often constrained more by diffusive loss [18, 59], competing reactions [6, 19], intermediate instability [7, 61, 62], and kinetic imbalance [6, 18, 63-65] than by individual enzyme activity.

Nature addresses this challenge through substrate channeling [7, 62], a principle increasingly exploited in engineered multienzyme assemblies [18, 21, 59, 65]. Mechanistically, substrate channeling broadly refers to the preferential transfer of intermediates between sequential catalytic sites, whereas substrate tunneling denotes a structurally specialized subset in which intermediates traverse physically continuous, enclosed molecular tunnels [18, 59, 61, 62]. The CODH–ACS complex exemplifies this specialized subset of substrate channeling, in which CO is transferred through a continuous molecular tunnel linking the CODH and ACS active sites [34-36, 47]. Without diffusing into the bulk solution, this protected pathway minimizes intermediate loss and competing reactions [18, 21, 59, 62], demonstrating how coordinated intermediate transfer improves pathway efficiency. Carrier-mediated biosynthetic systems further illustrate this principle [66, 67]. Acyl carrier proteins in fatty acid synthases shuttle covalently tethered intermediates between catalytic domains through coordinated conformational dynamics [68-70], whereas peptidyl carrier proteins in nonribosomal peptide synthetases mediate analogous transfers through transient, domain-specific interactions [67, 71-73]. Likewise, lipoyl domains in pyruvate dehydrogenase complexes function as molecular swinging arms [74-76], linking spatially separated active sites while maintaining high local concentrations of reaction intermediates [76-78]. These systems avoid passive diffusion by mediating efficient intermediate transfer through dynamic protein-protein interactions [62, 66, 67, 74] that direct intermediates from one catalytic site to the next.

Inspired by these naturally evolved mechanisms, protein scaffolds [63, 65], enzyme fusions [60, 65], and artificial metabolons [58, 60, 65] increase effective local enzyme concentration, reduce intermediate diffusion, and facilitate efficient intermediate transfer without redesigning individual enzymes. Synthetic enzyme and whole-cell CO-to-formate cascades further validate this engineering principle [46, 79, 80]. In these cascades, CODH first oxidizes CO to CO_2_, followed by formate dehydrogenase (FDH)-catalyzed reduction of CO_2_ to formate [46, 80]. Successful cascade construction required coordinated optimization of the two catalytic reactions, enzyme compatibility, redox balance, and reaction conditions [46, 79-81], rather than optimization of either enzyme alone. Systematic evaluation of multiple CODHs and FDHs prior to cascade construction further demonstrated that compatibility between sequential catalytic steps, rather than individual enzyme activity, governs overall cascade performance [46, 79, 80]. As a result, these examples support coordinated molecular transfer as a general design principle for improving pathway efficiency. Within the proposed framework, the CO-to-formate cascades exemplify this strategy by showing how efficient coordination between sequential catalytic modules determines overall cascade performance. Efficient intermediate transfer is therefore critical for pathway-level performance (Fig. 1). More broadly, pathway efficiency often depends on molecular compatibility between successive catalytic modules.

### Improve systems-level biocatalysis by engineering molecular exchange

As microbial biocatalysis advances from individual enzymes and multienzyme assemblies to whole-cell biocatalysts and microbial cell factories [3, 12, 14, 19], engineering challenges progressively shift from molecular entry and intermediate transfer to molecular exchange across biological systems. Sustained catalysis requires coordinated exchange of substrates and products across membranes [17, 82, 83], gases across cellular and gas–liquid interfaces [46, 79, 80, 84], electrons across catalytic interfaces [37-39], and cofactors across metabolic networks [3, 19, 64]. Molecular accessibility thus emerges as a systems-level design principle governing overall biocatalytic performance.

Electroenzymatic systems illustrate this transition [37, 38]. Although FDHs, hydrogenases, and CODHs possess outstanding catalytic activities [28, 47, 48], overall performance is frequently limited by interfacial and intramolecular electron-transfer pathways rather than catalytic turnover [38, 39, 85-87]. Engineering electrode architecture [37-39], enzyme orientation [38, 85], mediator accessibility [37, 81, 85], and electron-transfer distance [38, 39, 87] improves current density, Faradaic efficiency, and operational stability without modifying catalytic chemistry. For example, an O_2_-tolerant Y94S variant of FDH from *Shewanella oneidensis* MR-1 enabled direct bioelectrocatalytic CO_2_ reduction, producing 2.88 ± 0.03 mmol formate over 64 h at a steady rate of 45.3 ± 0.5 μmol h^–1^ cm^–2^ with a Faradaic efficiency of 93.1 ± 5.2% [87]. This example indicates that engineering electron exchange improves systems-level biocatalysis without altering catalytic chemistry. Accordingly, electroenzymatic systems exemplify molecular exchange, in which electron accessibility determines overall bioelectrocatalytic performance.

Accessibility constraints also arise in whole-cell biocatalysts, where membrane transport plays a central role in substrate uptake and product export before and after intracellular catalysis [17, 82, 83]. Engineering transport [17, 82, 83], intracellular cofactor availability [3, 19, 64], and gas-liquid mass transfer [46, 79, 80] often determine productivity, particularly for gaseous substrates such as CO, CO_2_, H2, and syngas. The same principle extends to microbial cell factories [3, 14, 19]. In a recent study, aromatic ester production reached approximately 10.4 g L^–1^, representing a ~4,700-fold improvement over the parental strain through the integration of tunnel engineering, dynamic pathway regulation, and metabolic rewiring [88]. Rather than arising solely from enzyme engineering, this improvement likely resulted from the coordinated optimization of substrate accessibility, acetyl-CoA supply, and intracellular precursor allocation [88].

Nature employs analogous strategies through bacterial microcompartments [89, 90], encapsulins [91], and biomolecular condensates [92, 93], which spatially organize catalytic functions while selectively regulating molecular exchange. Inspired by these natural systems, synthetic compartmentalization using engineered protein scaffolds [63, 65] and artificial organelles [90, 94, 95] improves pathway productivity by spatially controlling molecular accessibility. Collectively, molecular entry, transfer, and exchange form a multiscale engineering continuum for microbial biocatalysis. These systems demonstrate that molecular exchange contributes to compatibility between biocatalysts and their surrounding biological and electrochemical environments. As biological complexity increases, productive molecular exchange increasingly influences the translation of catalytic potential into overall biocatalytic performance (Fig. 1).

## Toward Molecular Access Engineering for Microbial Biocatalysis

Taken together, these examples demonstrate that molecular accessibility represents a distinct systems-level design dimension across microbial biocatalysis. Although these constraints arise from substrate entry, intermediate transfer, and molecular exchange, the corresponding engineering strategies share the engineering objective of improving molecular accessibility. Accordingly, molecular access engineering can be defined by the shared engineering objective of improving biocatalytic performance through rational control of molecular accessibility, rather than by any particular technology or biological system. Catalytic engineering determines what reactions are chemically possible, whereas molecular access engineering helps determine whether those reactions can be realized within integrated biological systems. Functional biocatalytic performance therefore emerges from the interplay between intrinsic catalytic potential and molecular accessibility, with the former defining reaction capability and the latter influencing how effectively that catalytic potential is translated into biological function. Together, catalytic engineering and molecular access engineering provide an integrated engineering framework for the rational engineering of advanced microbial biocatalysis.

As molecular accessibility emerges as a common contributor to functional compatibility across microbial biocatalytic systems, the next challenge is to develop predictive principles for its quantitative engineering [43, 44, 96]. Unlike catalytic activity, which is described by established kinetic parameters such as *k*_cat_, *K*_M_, and catalytic efficiency, this property still lacks quantitative descriptors that can predict compatibility across biological scales and link it to biocatalytic performance. Because molecular entry, molecular transfer, and molecular exchange arise from distinct biological mechanisms, no single descriptor is likely to capture all aspects of molecular accessibility. Instead, different accessibility-related processes will probably require complementary, mechanism-specific descriptors. Candidate descriptors may include molecular flux [97], effective diffusivity and transport-energy barriers [98, 99], tunnel openness, bottleneck geometry, and ligand-passage energetics [43, 44, 96-99], membrane permeability and transport efficiency [17, 82, 83], and interfacial electron-transfer efficiency [38], although no unified framework has yet integrated these parameters across biological scales. Future efforts should integrate these descriptors into multiscale predictive models [100]. Such integration could help transform molecular access engineering into a more predictive engineering discipline by linking molecular accessibility to biocatalytic performance. At present, however, the proposed framework remains conceptual and is intended to organize accessibility-related engineering strategies, not to serve as a comprehensive predictive model. Ultimately, such a framework may provide a common language for comparing and engineering microbial biocatalytic systems.

Future progress is likely to depend on integrating molecular accessibility across biological scales rather than optimizing each level in isolation. Because accessibility constraints can propagate across enzymes, catalytic assemblies, biological interfaces, and cellular metabolism, improvements achieved at one level may be constrained by another. Recent advances in intracellular flux prediction, automated biofoundry-integrated platforms, and deep learning-guided biological design highlight the growing potential of predictive and data-driven strategies to connect molecular, cellular, and process-level engineering [101-103]. These distinct design dimensions expand opportunities to improve oxygen tolerance, pathway efficiency, mass-transfer compatibility, and systems productivity while broadening the design space for microbial biocatalysis. Across biological scales, molecular entry, transfer, and exchange can be viewed as forming a hierarchical continuum through which catalytic potential is translated into biocatalytic performance. Achieving this goal will require integrating structural biology, molecular simulations, quantitative transport measurements, and data-driven multiscale predictive modeling to establish molecular access engineering as a practical and predictive design principle for future microbial biocatalysts [100].

## Conclusion

Microbial biocatalysis has advanced through improvements in catalytic activity enabled by enzyme engineering, metabolic engineering, and synthetic biology. This Review presents a complementary perspective in which functional biocatalytic performance is shaped not only by catalytic potential but also by molecular accessibility. Across diverse microbial biocatalytic platforms, including gas-converting enzymes, multienzyme assemblies, electroenzymatic systems, whole-cell biocatalysts, and microbial cell factories, similar accessibility-related constraints emerge despite substantial differences in biological organization, highlighting molecular accessibility as a shared engineering consideration spanning biological scales. Rather than proposing yet another biocatalyst-engineering methodology, molecular access engineering offers a general design principle to improve microbial biocatalysis through rational control of molecular accessibility. Engineering molecular entry, coordinating molecular transfer, and optimizing molecular exchange represent complementary strategies that collectively contribute to a common objective: maximizing productive molecular interactions with catalytic functions. In this perspective, catalytic engineering defines catalytic potential, whereas molecular access engineering influences how efficiently that potential is realized. These distinct design dimensions expand the design space for developing robust, selective, and scalable microbial biocatalytic systems. Future advances in microbial biocatalysis are likely to benefit from integrating catalytic engineering with molecular access engineering.

## Figures and Tables

**Table 1:** Molecular access engineering across microbial biocatalytic platforms.

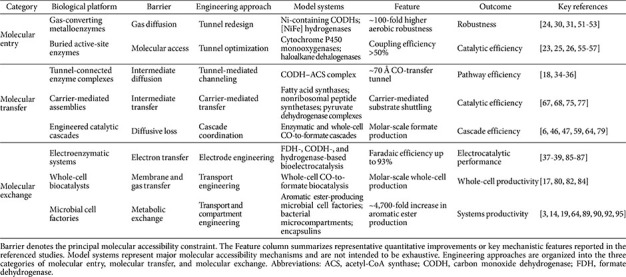

## References

[1] Bornscheuer UT, Huisman GW, Kazlauskas RJ, Lutz S, Moore JC, Robins K (2012). Engineering the third wave of biocatalysis. Nature.

[2] Sheldon RA, Woodley JM (2018). Role of biocatalysis in sustainable chemistry. Chem. Rev..

[3] Nielsen J, Keasling JD (2016). Engineering cellular metabolism. Cell.

[4] Lee SY, Kim HU, Chae TU, Cho JS, Kim JW, Shin JH (2019). A comprehensive metabolic map for production of bio-based chemicals. Nat. Catal..

[5] Choi KR, Lee SY (2023). Systems metabolic engineering of microorganisms for food and cosmetics production. Nat. Rev. Bioeng..

[6] Dueber JE, Wu GC, Malmirchegini GR, Moon TS, Petzold CJ, Ullal AV (2009). Synthetic protein scaffolds provide modular control over metabolic flux. Nat. Biotechnol..

[7] Sweetlove LJ, Fernie AR (2018). The role of dynamic enzyme assemblies and substrate channelling in metabolic regulation. Nat. Commun..

[8] Arnold FH (2018). Directed evolution: bringing new chemistry to life. Angew. Chem. Int. Ed..

[9] Huang PS, Boyken SE, Baker D (2016). The coming of age of de novo protein design. Nature.

[10] Way JC, Collins JJ, Keasling JD, Silver PA (2014). Integrating biological redesign: where synthetic biology came from and where it needs to go. Cell.

[11] Khalil AS, Collins JJ (2010). Synthetic biology: applications come of age. Nat. Rev. Genet..

[12] Kissman EN, Sosa MB, Millar DC, Koleski EJ, Thevasundaram K, Chang MCY (2024). Expanding chemistry through *in vitro* and *in vivo* biocatalysis. Nature.

[13] Bayer T, Wu S, Snajdrova R, Baldenius K, Bornscheuer UT (2025). An update: enzymatic synthesis for industrial applications. Angew. Chem. Int. Ed..

[14] Kim GB, Kim HR, Lee SY (2025). Comprehensive evaluation of the capacities of microbial cell factories. Nat. Commun..

[15] Hauer B (2020). Embracing naturés catalysts: a viewpoint on the future of biocatalysis. ACS Catal..

[16] Woodley JM (2008). New opportunities for biocatalysis: making pharmaceutical processes greener. Trends Biotechnol..

[17] Lv X, Xue H, Qin L, Li C (2022). Transporter engineering in microbial cell factory boosts biomanufacturing capacity. Biodes. Res..

[18] Wheeldon I, Minteer SD, Banta S, Barton SC, Atanassov P, Sigman M (2016). Substrate channelling as an approach to cascade reactions. Nat. Chem..

[19] Gianolio S, Mrigwani A, Paradisi F (2025). Advances in integrating microbial metabolism with catalytic systems. Nat. Chem. Biol..

[20] Lovelock SL, Crawshaw R, Basler S, Levy C, Baker D, Hilvert D (2022). The road to fully programmable protein catalysis. Nature.

[21] Cao Y, Qiao W, Zhang C, Zhu M, Wu Q, Lou W-Y (2025). Programmable enzyme catalysis based on multiscale confinements. Nat. Synth..

[22] Woodley JM (2019). Accelerating the implementation of biocatalysis in industry. Appl. Microbiol. Biotechnol..

[23] Kokkonen P, Bednar D, Pinto G, Prokop Z, Damborsky J (2019). Engineering enzyme access tunnels. Biotechnol. Adv..

[24] Kim SM, Kang SH, Jeon BW, Kim YH (2024). Tunnel engineering of gas-converting enzymes for inhibitor retardation and substrate acceleration. Bioresour. Technol..

[25] Gora A, Brezovsky J, Damborsky J (2013). Gates of enzymes. Chem. Rev..

[26] Brezovsky J, Babkova P, Degtjarik O, Fortova A, Gora A, Iermak I (2016). Engineering a de novo transport tunnel. ACS Catal..

[27] Jeoung JH, Dobbek H (2007). Carbon dioxide activation at the Ni,Fe-cluster of anaerobic carbon monoxide dehydrogenase. Science.

[28] Stripp ST, Duffus BR, Fourmond V, Leger C, Leimkuhler S, Hirota S (2022). Second and outer coordination sphere effects in nitrogenase, hydrogenase, formate dehydrogenase, and CO dehydrogenase. Chem. Rev..

[29] Svetlitchnyi V, Peschel C, Acker G, Meyer O (2001). Two membrane-associated NiFeS-carbon monoxide dehydrogenases from the anaerobic carbon-monoxide-utilizing eubacterium *Carboxydothermus hydrogenoformans*. J. Bacteriol..

[30] Kim SM, Lee J, Kang SH, Heo Y, Yoon HJ, Hahn JS (2022). O_2_-tolerant CO dehydrogenase via tunnel redesign for the removal of CO from industrial flue gas. Nat. Catal..

[31] Kim SM, Kong SY, Kang J, Ji JS, Kang SH, Yoon HJ (2025). Fortification of FeS clusters reshapes anaerobic CO dehydrogenase into an air-viable enzyme through multilayered sealing of O_2_ tunnels. Angew. Chem. Int. Ed..

[32] Chovancova E, Pavelka A, Benes P, Strnad O, Brezovsky J, Kozlikova B (2012). CAVER 3.0: a tool for the analysis of transport pathways in dynamic protein structures. PLoS Comput. Biol..

[33] Pavelka A, Sebestova E, Kozlikova B, Brezovsky J, Sochor J, Damborsky J (2016). CAVER: algorithms for analyzing dynamics of tunnels in macromolecules. IEEE Trans. Comput. Biol. Bioinform..

[34] Doukov TI, Iverson TM, Seravalli J, Ragsdale SW, Drennan CL (2002). A Ni-Fe-Cu center in a bifunctional carbon monoxide dehydrogenase/acetyl-CoA synthase. Science.

[35] Tan X, Loke HK, Fitch S, Lindahl PA (2005). The tunnel of acetyl-coenzyme A synthase/carbon monoxide dehydrogenase regulates delivery of CO to the active site. J. Am. Chem. Soc..

[36] Wang PH, Bruschi M, De Gioia L, Blumberger J (2013). Uncovering a dynamically formed substrate access tunnel in carbon monoxide dehydrogenase/acetyl-CoA synthase. J. Am. Chem. Soc..

[37] Ruff A (2017). Redox polymers in bioelectrochemistry: common playgrounds and novel concepts. Curr. Opin. Electrochem..

[38] Lee H, Reginald SS, Sravan JS, Lee M, Chang IS (2025). Advanced strategies for enzyme-electrode interfacing in bioelectrocatalytic systems. Trends Biotechnol..

[39] Becker JM, Lielpetere A, Szczesny J, Junqueira JRC, Rodriguez-Macia P, Birrell JA (2022). Bioelectrocatalytic CO_2_ reduction by redox polymer-wired carbon monoxide dehydrogenase gas diffusion electrodes. ACS Appl. Mater. Interfaces.

[40] Sheldon RA, Brady D (2018). The limits to biocatalysis: pushing the envelope. Chem. Commun. (Camb.).

[41] Bommarius AS, Paye MF (2013). Stabilizing biocatalysts. Chem. Soc. Rev..

[42] Bloom JD, Labthavikul ST, Otey CR, Arnold FH (2006). Protein stability promotes evolvability. Proc. Natl. Acad. Sci. USA.

[43] Kingsley LJ, Lill MA (2015). Substrate tunnels in enzymes: Structure-function relationships and computational methodology. Proteins.

[44] Prokop Z, Gora A, Brezovsky J, Koudelakova T, Stepankova V, Damborsky J. 2012. Engineering of protein tunnels: keyhole-lock-key model for catalysis by the enzymes with buried active sites, pp. 421–464. *In* Lutz S, Bornscheuer UT (eds.), *Protein Engineering Handbook*, Vol. 1, 2nd ed. Wiley-VCH, Weinheim, Germany.

[45] Kim SM, Kim YH (2024). Streamlined aromatic ester process via tunnel engineering. Nat. Chem. Eng..

[46] Lee J, Kim SM, Jeon BW, Hwang HW, Poloniataki EG, Kang J (2024). Molar-scale formate production via enzymatic hydration of industrial off-gases. Nat. Chem. Eng..

[47] Can M, Armstrong FA, Ragsdale SW (2014). Structure, function, and mechanism of the nickel metalloenzymes, CO dehydrogenase, and acetyl-CoA synthase. Chem. Rev..

[48] Arriaza-Gallardo FJ, Zheng YC, Gehl M, Nomura S, Fernandes-Queiroz JP, Shima S (2023). [Fe]-hydrogenase, cofactor biosynthesis and engineering. ChemBioChem.

[49] Dobbek H, Svetlitchnyi V, Gremer L, Huber R, Meyer O (2001). Crystal structure of a carbon monoxide dehydrogenase reveals [Ni–4Fe–5S] cluster. Science.

[50] Dementin S, Leroux F, Cournac L, de Lacey AL, Volbeda A, Leger C (2009). Introduction of methionines in the gas channel makes [NiFe] hydrogenase aero-tolerant. J. Am. Chem. Soc..

[51] Liebgott PP, Leroux F, Burlat B, Dementin S, Baffert C, Lautier T (2010). Relating diffusion along the substrate tunnel and oxygen sensitivity in hydrogenase. Nat. Chem. Biol..

[52] Leroux F, Dementin S, Burlat B, Cournac L, Volbeda A, Champ S (2008). Experimental approaches to kinetics of gas diffusion in hydrogenase. Proc. Natl. Acad. Sci. USA.

[53] Duché O, Elsen S, Cournac L, Colbeau A (2005). Enlarging the gas access channel to the active site renders the regulatory hydrogenase HupUV of *Rhodobacter capsulatus* O_2_ sensitive without affecting its transductory activity. FEBS J..

[54] Meng S, An R, Li Z, Schwaneberg U, Ji Y, Davari MD (2021). Tunnel engineering for modulating the substrate preference in cytochrome P450_Bsβ_HI. Bioresour. Bioprocess.

[55] Meng S, Ji Y, Liu L, Davari MD, Schwaneberg U (2022). Modulating the coupling efficiency of P450 BM3 by controlling water diffusion through access tunnel engineering. ChemSusChem.

[56] Biedermannova L, Prokop Z, Gora A, Chovancova E, Kovacs M, Damborsky J (2012). A single mutation in a tunnel to the active site changes the mechanism and kinetics of product release in haloalkane dehalogenase LinB. J. Biol. Chem..

[57] Kokkonen P, Beier A, Mazurenko S, Damborsky J, Bednar D, Prokop Z (2021). Substrate inhibition by the blockage of product release and its control by tunnel engineering. RSC Chem. Biol..

[58] Artzi L, Bayer EA, Morais S (2017). Cellulosomes: bacterial nanomachines for dismantling plant polysaccharides. Nat. Rev. Microbiol..

[59] Zhang YH (2011). Substrate channeling and enzyme complexes for biotechnological applications. Biotechnol. Adv..

[60] DeLisa MP, Conrado RJ (2009). Synthetic metabolic pipelines. Nat. Biotechnol..

[61] Miles EW, Rhee S, Davies DR (1999). The molecular basis of substrate channeling. J. Biol. Chem..

[62] Huang X, Holden HM, Raushel FM (2001). Channeling of substrates and intermediates in enzyme-catalyzed reactions. Annu. Rev. Biochem..

[63] Conrado RJ, Wu GC, Boock JT, Xu H, Chen SY, Lebar T (2012). DNA-guided assembly of biosynthetic pathways promotes improved catalytic efficiency. Nucleic Acids Res..

[64] Ream M, Prather KLJ (2024). Engineered autonomous dynamic regulation of metabolic flux. Nat. Rev. Bioeng..

[65] Chen Z, Wu T, Yu S, Li M, Fan X, Huo YX (2024). Self-assembly systems to troubleshoot metabolic engineering challenges. Trends Biotechnol..

[66] Buyachuihan L, Stegemann F, Grininger M (2024). How acyl carrier proteins (ACPs) direct fatty acid and polyketide biosynthesis. Angew. Chem. Int. Ed..

[67] Kittilä T, Mollo A, Charkoudian LK, Cryle MJ (2016). New structural data reveal the motion of carrier proteins in nonribosomal peptide synthesis. Angew. Chem. Int. Ed..

[68] Schultz K, Costa-Pinheiro P, Gardner L, Pinheiro LV, Ramirez-Solis J, Gardner SM (2025). Snapshots of acyl carrier protein shuttling in human fatty acid synthase. Nature.

[69] Singh K, Bunzel G, Graf B, Yip KM, Neumann-Schaal M, Stark H (2023). Reconstruction of a fatty acid synthesis cycle from acyl carrier protein and cofactor structural snapshots. Cell.

[70] Choi W, Li C, Chen Y, Wang Y, Cheng Y (2025). Structural dynamics of human fatty acid synthase in the condensing cycle. Nature.

[71] Reimer JM, Haque AS, Tarry MJ, Schmeing TM (2018). Piecing together nonribosomal peptide synthesis. Curr. Opin. Struct. Biol..

[72] Heberlig GW, La Clair JJ, Burkart MD (2025). Crosslinking intermodular condensation in non-ribosomal peptide biosynthesis. Nature.

[73] Pistofidis A, Ma P, Li Z, Munro K, Houk KN, Schmeing TM (2025). Structures and mechanism of condensation in non-ribosomal peptide synthesis. Nature.

[74] Perham RN (2000). Swinging arms and swinging domains in multifunctional enzymes: catalytic machines for multistep reactions. Annu. Rev. Biochem..

[75] de Kok A, Hengeveld AF, Martin A, Westphal AH (1998). The pyruvate dehydrogenase multi-enzyme complex from Gram-negative bacteria. Biochim. Biophys. Acta.

[76] Patel MS, Nemeria NS, Furey W, Jordan F (2014). The pyruvate dehydrogenase complexes: structure-based function and regulation. J. Biol. Chem..

[77] Skerlova J, Berndtsson J, Nolte H, Ott M, Stenmark P (2021). Structure of the native pyruvate dehydrogenase complex reveals the mechanism of substrate insertion. Nat. Commun..

[78] Wang C, Ma C, Xu Y, Chang S, Wu H, Yan C (2025). Dynamics of the mammalian pyruvate dehydrogenase complex revealed by in-situ structural analysis. Nat. Commun..

[79] Kim TH, Yun J, Lee JS, Lee MJ, Kim JK, Lee J (2025). Enhancing biological CO to formate conversion: application of aerophobic coatings to mitigate bubble coalescence. ACS Sustain. Chem. Eng..

[80] Kim TH, Lee JS, Lee J, Kim YH, Na JG, Oh BK (2026). Intercellular whole-cell cascade strategy for robust CO-to-formate bioconversion. Bioresour. Technol..

[81] Kim SM, Kang SH, Lee J, Heo Y, Poloniataki EG, Kang J (2024). Identifying a key spot for electron mediator-interaction to tailor CO dehydrogenase's affinity. Nat. Commun..

[82] van der Hoek SA, Borodina I (2020). Transporter engineering in microbial cell factories: the ins, the outs, and the in-betweens. Curr. Opin. Biotechnol..

[83] Cheng Y, Xie X, Guo L, Ji H, Madadi M, Qiao Y. 2026. Transporter engineering in microbial cell factories. *Trends Biotechnol.* Available online. https://doi.org/10.1016/j.tibtech.2026.06.010. 10.1016/j.tibtech.2026.06.010 42342469

[84] Wang D, Zhang H, Wang YK, Pinelo M, Mazzei R, Fan R (2025). Optimizing enzymatic bioreactors: the role of mass transfer in enhancing catalytic efficiency and stability. Chem. Eng. J..

[85] Liu W, Zhang K, Liu J, Wang Y, Zhang M, Cui H (2024). Bioelectrocatalytic carbon dioxide reduction by an engineered formate dehydrogenase from *Thermoanaerobacter kivui*. Nat. Commun..

[86] Liu Y, Webb S, Moreno-Garcia P, Kulkarni A, Maroni P, Broekmann P (2023). Facile functionalization of carbon electrodes for efficient electroenzymatic hydrogen production. JACS AU.

[87] Liu WS, Zhang P, Wang XF, Zhang KC, Yang WH, Cui HJ (2026). An interfacial-intramolecular electron highway for accelerated electrocatalytic CO_2_ reduction by an O_2_-tolerant formate dehydrogenase. Nat. Commun..

[88] Lu L, Wang X, Wang T, Shen X, Sun X, Tian P (2024). A bacterial platform for producing aromatic esters from glycerol. Nat. Chem. Eng..

[89] Kerfeld CA, Aussignargues C, Zarzycki J, Cai F, Sutter M (2018). Bacterial microcompartments. Nat. Rev. Microbiol..

[90] Doron L, Kerfeld CA (2024). Bacterial microcompartments as a next-generation metabolic engineering tool: utilizing nature's solution for confining challenging catabolic pathways. Biochem. Soc. Trans..

[91] Nichols RJ, Cassidy-Amstutz C, Chaijarasphong T, Savage DF (2017). Encapsulins: molecular biology of the shell. Crit. Rev. Biochem. Mol. Biol..

[92] Banani SF, Lee HO, Hyman AA, Rosen MK (2017). Biomolecular condensates: organizers of cellular biochemistry. Nat. Rev. Mol. Cell Biol..

[93] Hodgins L, Parmar BS, Reyes-Lamothe R, Weber SC (2026). Size matters: a biophysical perspective on biomolecular condensates in bacteria. Annu. Rev. Biophys..

[94] Fang Z, Zhu YJ, Qian ZG, Xia XX (2024). Designer protein compartments for microbial metabolic engineering. Curr. Opin. Biotechnol..

[95] Lau YH, Giessen TW, Altenburg WJ, Silver PA (2018). Prokaryotic nanocompartments form synthetic organelles in a eukaryote. Nat. Commun..

[96] Mandal N, Stevens JA, Poma AB, Surpeta B, Sequeiros-Borja C, Thirunavukarasu AS (2026). Unlocking high-throughput investigation of transport tunnels in enzymes using coarse-grained simulation methods. J. Chem. Theory Comput..

[97] Brezovsky J, Thirunavukarasu AS, Surpeta B, Sequeiros-Borja CE, Mandal N, Sarkar DK (2022). TransportTools: a library for high-throughput analyses of internal voids in biomolecules and ligand transport through them. Bioinformatics.

[98] Vavra O, Damborsky J, Bednar D (2022). Fast approximative methods for study of ligand transport and rational design of improved enzymes for biotechnologies. Biotechnol. Adv..

[99] Torabifard H, Cisneros GA (2017). Computational investigation of O_2_ diffusion through an intra-molecular tunnel in AlkB; influence of polarization on O_2_ transport. Chem. Sci..

[100] Peng GCY, Alber M, Tepole AB, Cannon WR, De S, Dura-Bernal S (2021). Multiscale modeling meets machine learning: what can we learn?. Arch. Comput. Methods Eng..

[101] Bae SH, Sim MS, Jeong KJ, He D, Kwon I, Kim TW (2024). Intracellular flux prediction of recombinant *Escherichia coli* producing gamma-aminobutyric acid. J. Microbiol. Biotechnol..

[102] Jun JS, Hong S, Park JH, Shin J, Lee DH (2025). Automated and programmable cell-free systems for scalable synthetic biology with a focus on biofoundry integration. J. Microbiol. Biotechnol..

[103] Seo E, Sung D, Lee JW (2025). Deep generative model-driven design of microbial synthetic promoters. J. Microbiol. Biotechnol..

